# Effects of Hazelnut Consumption on Blood Lipids and Body Weight: A Systematic Review and Bayesian Meta-Analysis

**DOI:** 10.3390/nu8120747

**Published:** 2016-11-25

**Authors:** Simone Perna, Attilio Giacosa, Gianluca Bonitta, Chiara Bologna, Antonio Isu, Davide Guido, Mariangela Rondanelli

**Affiliations:** 1 Department of Public Health, Experimental and Forensic Medicine, Endocrinology and Nutrition Unit, University of Pavia, Azienda di Servizi alla Persona di Pavia, Pavia 27100, Italy; chiara.bologna02@universitadipavia.it (C.B.); antonio.isu@gmail.com (A.I.); mariangela.rondanelli@unipv.it (M.R.); 2 Department of Gastroenterology, Policlinico di Monza, Monza 20097, Italy; attilio.giacosa@policlinicodimonza.it; 3 Department of Biomedical Sciences for Health, Division of General Surgery, IRCCS Policlinico San Donato, University of Milan, Milan 20097, Italy; bbonit@icloud.com; 4 Department of Public Health, Experimental and Forensic Medicine, Biostatistics and Clinical Epidemiology Unit, University of Pavia, Pavia 27100, Italy; davide.guido@unipv.it

**Keywords:** hazelnut, lipid, cholesterol, triglyceride, obesity, body weight, BMI, Corylus avellana

## Abstract

Hazelnuts are rich in monounsaturated fatty acids and antioxidant bioactive substances: their consumption has been associated with a decreased risk of cardiovascular disease events. A systematic review and a meta-analysis was performed to combine the results from several trials and to estimate the pooled (overall) effect of hazelnuts on blood lipids and body weight outcomes. Specifically, a Bayesian random effect meta-analysis of mean differences of Δ-changes from baseline across treatment (MDΔ) (i.e., hazelnut-enriched diet vs. control diet) has been conducted. Nine studies representing 425 participants were included in the analysis. The intervention diet lasted 28–84 days with a dosage of hazelnuts ranging from 29 to 69 g/day. Out of nine studies, three randomized studies have been meta-analyzed showing a significant reduction in low-density lipoprotein (LDL) cholesterol (pooled MDΔ = −0.150 mmol/L; 95% highest posterior density interval (95%HPD) = −0.308; −0.003) in favor of a hazelnut-enriched diet. Total cholesterol showed a marked trend toward a decrease (pooled MDΔ = −0.127 mmol/L; 95%HPD = −0.284; 0.014) and high-density lipoprotein (HDL) cholesterol remained substantially stable (pooled MDΔ = 0.002 mmol/L; 95%HPD = −0.140; 0.147). No effects on triglycerides (pooled MDΔ = 0.045 mmol/L; 95%HPD = −0.195; 0.269) and body mass index (BMI) (pooled MDΔ = 0.062 kg/m^2^; 95%HPD = −0.293; 0.469) were found. Hazelnut-enriched diet is associated with a decrease of LDL and total cholesterol, while HDL cholesterol, triglycerides and BMI remain substantially unchanged.

## 1. Introduction

The relationship between the consumption of nuts and risk of cardiovascular disease (CVD) has been a major research interest in the recent years. There is a large body of epidemiologic and intervention studies that demonstrate the multiple beneficial effects of nut consumption on CVD prevention [1]. In particular, walnuts, almonds, peanuts and pecan nuts show a significant lowering effect on total serum cholesterol (TC) and low-density lipoprotein cholesterol (LDL) [2].

Recent clinical trials have shown that also hazelnuts play a favorable role on plasma lipid pattern changes and could therefore favor the reduction of CVD morbidity and mortality [3,4,5,6,7,8,9,10].

Hazelnuts may be associated with CVD prevention because of their fatty acid composition, mostly based on monounsaturated fatty acids (MUFA), that protect low-density lipoproteins (LDL) against oxidation. Moreover, hazelnuts are rich in various bioactive substances such as tocopherols and phytosterols, l-arginine [10,11], selenium, caffeic acid, fibers [12,13], gallic acid, p-hydroxy benzoic acid, epicatechin, sinapic acid and quercetin that could have anti-atherogenic effects by means of biological mechanisms acting on various pathways in CVD development [14].

Various clinical studies have been performed on the effect of hazelnut intake on human serum lipids, but as of yet no review or meta-analysis of the existing data has been published. In the current study, we conducted a systematic evaluation of the existing clinical trials to review the effects of hazelnut consumption on blood lipid levels (cholesterol, triglycerides, apolipoprotein A and B) as well as on body weight. We also performed a Bayesian random effect meta-analysis on the following outcomes: total cholesterol, HDL, LDL, triglycerides and body mass index (BMI). Body weight and BMI were considered because of the existing concern about supplementation with a product which is rich in calories and could therefore be associated with a negative outcome, i.e., body weight increase.

## 2. Experimental Section

### 2.1. Data Sources and Search Strategy

The present systematic review was conducted in accordance with the PRISMA (Preferred Reporting Items for Systematic Review and Meta-Analyses) statement [15]. The search involved all the studies published from the 1st of January 1958 to the 31st of October 2015. English-written articles were identified by searching the Medline database [16], Scopus [17], ISI Web of Science [18], and Google Scholar [19]. The search strategy was based on the following items: hazelnuts AND “lipoproteins” OR “cholesterol” OR “triglyceride” OR “lipoprotein” OR “lipid profile” OR “TG” OR “TC” OR “blood lipids” OR “APO” OR “apolipoprotein” OR “CVD” OR “disease” OR “body weight” OR “BMI” OR “Body mass index” OR “cardiovascular”.

### 2.2. Inclusion and Exclusion Criteria

Two authors (SP; MR) independently reviewed each report. For each of the relevant abstracts, full publications were retrieved for evaluation on the basis of criteria established a priori. We included original clinical trials investigating pre- versus post-administration effects of a hazelnut-enriched diet and its eventual comparison with a control diet (if available), when at least one blood lipid marker was reported in healthy participants, or in patients with cardiovascular risk factors, including overweight, metabolic syndrome, obesity, hypercholesterolemia or diabetes. Additionally, the eligible studies needed to show hazelnut-based intervention with clear information about the amount and frequency of hazelnut supply and information about the participant’s diet. All adult-aged groups were included. The eligible studies were required to report baseline and follow-up values, the mean change (Δ-change) from baseline, and/or the mean difference Δ-changes among intervention groups for at least one lipid outcome.

Trials were excluded if they did not report the effects on blood lipid markers, or if a hazelnut-enriched diet was associated with the supplementation of other foods. For studies with more than one comparison group, we included in the study analysis only data regarding the hazelnut intervention group and the control group [7].

### 2.3. Data Collection

Study characteristics, which included authors, publication year, specific study design (single group, randomized, parallel/crossover), age, gender, baseline weight or BMI, were extracted for quantitative and qualitative analysis. In order to define the type of intervention, we focused on the specific amount of hazelnuts (percentage of energy due to hazelnuts or grams per day of hazelnuts), on the number of patients (control and/or intervention), on the number of supplementation days and on the type of control diet. We were interested in the following primary outcomes: mean serum total cholesterol (TC), low-density lipoprotein cholesterol (LDL), high-density lipoprotein cholesterol (HDL), triglycerides (TG), apolipoprotein A (APO-A) and B (APO-B). Moreover, the following secondary outcomes were considered: body weight and body mass index (BMI). The Δ-changes from baseline and/or the mean difference Δ-changes between intervention groups were the endpoints.

### 2.4. Risk of Bias in Individual Studies

The risk of bias of each study was assessed by two reviewers (SP and MR) with the Cochrane Collaboration Risk of Bias tool [20]. The factors regarded as contributing to the study quality were the generation of the allocation sequence, allocation concealment, blinding, blinding outcome data, incomplete data and selective reporting. We classified these factors as low risk of bias, high risk of bias, or unclear risk of bias. Due to the fact that blinding is not possible in clinical trials with dietary interventions, we judged the quality of the studies on the basis of the other five items (generation of the allocation sequence, allocation concealment, blinding outcome data, incomplete outcome data and selective reporting). Studies with a low risk of bias for at least three items were regarded as good; studies with a low risk of bias for at least two items were regarded as fair, and studies with a low risk for no item or only for one item were regarded as poor.

### 2.5. Bayesian Meta-Analysis

In addition to a systematic review, we performed a Bayesian random effect meta-analysis on the following outcomes: total cholesterol, HDL, LDL, triglycerides and BMI. For each study, we considered the mean Δ-changes from baseline for the hazelnut-enriched diet and control group. Accounting for this, the endpoint was the difference of mean Δ-changes (MDΔ) between treatments across time (post/pre). If more than one time point for follow-up was reported, we considered only the baseline value and the last one. Standard deviations (and/or standard errors) were also collected when possible. All serum lipid values were converted to mmol per liter, if necessary. 

Compared to traditional meta-analyses, we preferred the Bayesian approach [21] that takes into account all sources of variation and reflects these variations in the pooled result [22]. Furthermore, the Bayesian approach can provide more accurate estimates for small samples [23,24,25]. In this framework, full Bayesian analysis was performed using Markov chain Monte Carlo (MCMC) to fit the random effect meta-analysis model and get the pooled (overall) estimate (μ) of the endpoints (MDΔ) across studies. In addition, we also estimated and monitored two other relevant parameters, the between-study variance (τ^*2*^), i.e., the variability of MDΔ across studies, and the within-subject correlation (ρ) i.e., the intra-subject correlation between the outcome values before and after treatment, as suggested by Abrams et al. [26]. Accounting for MCMC algorithms, the results were based on 300,000 iterations after a burn-in period of 50,000 iterations. The accuracy of the results has been assessed by convergence of MCMC algorithm, that were checked both using graphical inspection of running means and using all methods included in R/Boa package [27]. The medians of the µ, τ^2^ and ρ posterior distribution (by MCMC) with respective 95% highest posterior density intervals (95%HPD) were computed and used as parameter estimates; the median of *µ* was taken as the pooled MDΔ. We judged the estimated parameters’ significance by whether its 95%HPD involved the value “0” [28]. In addition, standard deviations and Monte Carlo errors of posterior distributions were also computed to further validate the estimates.

Moreover, since we have worked in a Bayesian framework, we have assessed three different scenarios of prior distributions by recording all the parameter estimates. Consequently, we performed a sensitivity analysis by DIC (deviance information criterion) by trying a range of plausible prior distributions following Lambert et al. [29]. In this way, two vague priors and one weakly informative prior were elicited for the between-study variance (τ^*2*^) by the study precision (τ^2^)^−1^ or the standard deviation (τ): (i) an inverse gamma (0.001, 0.001) (model 1); (ii) a uniform (0,100) (model 2) and (iii) another uniform (0, 2) (model 3) (see Appendix).

Statistical analyses were performed by Jags [30], R [31] and its packages R2jag [32], boa [27] and coda [33].

## 3. Results

The literature search retrieved 779 papers. After screening, 385 papers were selected for full-text revision. After applying our inclusion and exclusion criteria, 376 studies were excluded and then nine clinical trials were selected for the present systematic review and meta-analysis.

**Figure 1:**
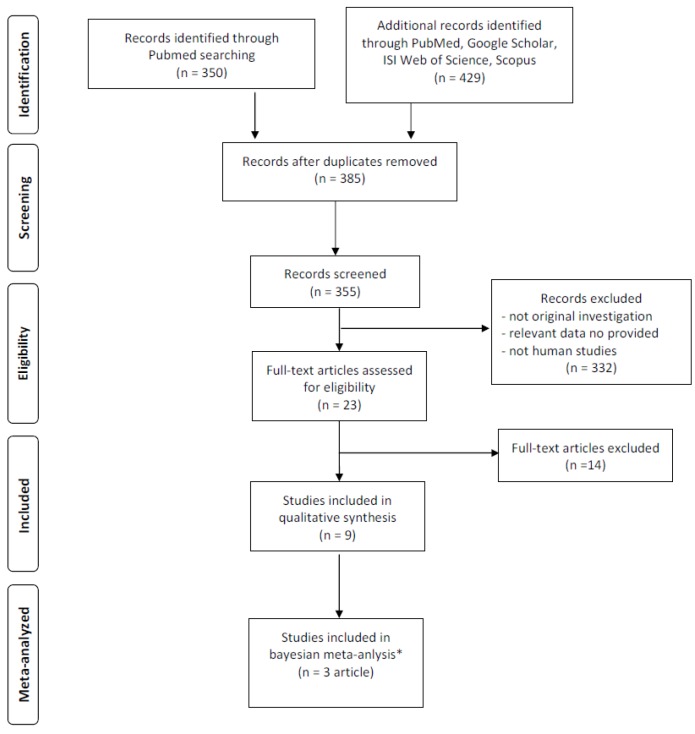
Study attrition diagram. * In Tey et al. (2013) [8] two hazelnut dosages (at 30 g/day and 60 g/day) compared with a control diet were administered. Accounting for this, we considered them as two separate studies.

These nine clinical trials studied a total of 425 adults: 195 (46%) were males and 230 (54%) were females, aged 18 to 55 years. Figure 1 shows the study selection process. The study characteristics are outlined in Table 1. Regarding the study design and the relative level of evidence (as suggested by the Centre for Evidence-Based Medicine [34]), out of the nine studies, five are randomized trials: one has a cross-over design, three have a parallel-study design and one is a controlled trial. The remaining four studies are non-randomized trials, three of which have a single-group study and one has a double-control sandwich model study. The baseline characteristics and comorbidity status of the participants varied (Table 1). Six studies recruited only healthy subjects, two studies recruited hypercholesterolemic subjects, and the remaining one involved only subjects with type 2 diabetes. Table 2 shows the inclusion and exclusion criteria for each study, including health and disease status. Dietary interventions lasted from 28 to 84 days (mean: 56 days) with a dosage of hazelnuts ranging from 29 to 69 g/days (mean: 45 g/day). Three papers have a control group identified by a self-managed diet without any type of nut. Nine studies (425 participants) reported results for serum TC, HDL-C, LDL-C and TG. Six studies (249 participants) reported results for serum Apo A and Apo B. Eight studies reported BMI data.

In Table 3, only the mean Δ-changes of the hazelnut intervention groups (and their statistical significances) are reported. Out of nine studies, a significant decrease (*p* < 0.05) in TC in four studies [4,6,10,35], in the order of −0.19, −0.20, −0.47 and −0.36 mmol/L, were found. The same four studies [4,6,10,35] showed a significant decrease (*p* < 0.05) of LDL-C in the order of −0.22, −0.36, −0.25, −0.21 mmol/L. Four studies [4,5,6,35] found a significant increase (*p* < 0.05) in HDL-C in order of 0.09, 0.07, 0.03 and 0.15 mmol/L. Two studies [5,35] found a significant decrease (*p* < 0.05) in TG in the order of −0.27 and −0.45 mmol/L, and one study reported an increase of TG [4]. Two studies [10,35] out of six found a significant increase (*p* < 0.05) in ApoA in the order of 0.15 and 0.06 g/L, and three studies [5,6,10] showed a significant decrease (*p* < 0.05) in Apo B in the order of −0.07, −0.04 and −0.07 g/L, respectively.

Out of eight studies with BMI data, six showed absence of difference, and one [35] showed a significant decrease (*p* < 0.05) of −0.5 kg/m^2^, and one showed an increase of BMI and body weight [7].

## 4. Risk of Bias

Table 4 shows the methodologic quality of the studies. None of the studies was suitable for all of the six items considered for the methodologic quality assessment because none of them was blind. A total of 44% of studies was rated as appropriate [6,7,8,9], whereas the method used to generate the allocation sequence was unclear in the others [4,5,10,35,36]. Allocation concealment and selective reporting were appropriate in 89% of the studies. In contrast, all of studies had a high risk of bias for blinding outcome assessment. All studies had no risk of bias for incomplete reporting. The overall quality was assessed and rated as “good” (low risk of bias) for four studies [6,7,8,9], “fair” for four studies [5,10,35,36], and “poor” for one study [4].

### Bayesian Meta-Analysis

According to the selected methodology, only three out of nine studies provided the minimum findings suitable for the Bayesian meta-analysis, i.e., Damavandi et al. [36], Tey et al. (2011) [7] and the two hazelnut dosages (at 30 g/day and 60 g/day, compared with a control group) administered in Tey et al. (2013) [8]. Apolipoprotein A and B have been excluded from the meta-analysis because these data were not reported in these studies.

Table 5 shows the meta-analysis data, while Table 6 displays the results by outcome, i.e., parameter estimates and sensitivity analysis. Model 1 is always preferred across the outcomes since DIC is the lowest; however, the MCMC medians of the pooled (overall) MDΔ (μ) are very similar across models. Notably, the standard deviations, 95%HPDs and MC errors in models 2 and 3 (τ prior—uniform) are always bigger than in model 1 where the τ^2^ prior distribution is an inverse gamma. Moreover, the τ^2^ and ρ 95%HPDs of model 1 are the most narrow across outcomes. This confirms the adequacy of model 1. In any event, concerning this model, from the four randomized trials meta-analyzed, we achieved a significant pooled reduction in LDL cholesterol (MDΔ = −0.150 mmol/L; 95% highest posterior density interval [95%HPD] = −0.308; −0.003) in favor of the hazelnut-enriched diet. The term “significant” indicates that MDΔ = 0 is not included in 95%HPD. Total cholesterol decreases (MDΔ = −0.127 mmol/L; 95%HPD = −0.284; 0.014) even though the positive value of one of the two HPD data appears above “0”. HDL cholesterol remains substantially stable (MDΔ = 0.002 mmol/L; 95%HPD = −0.140; 0.147). In addition, no effects on triglycerides (MDΔ = 0.045 mmol/L; 95%HPD = −0.195; 0.269) and body mass index (MDΔ = 0.062 kg/m^2^; 95%HPD = −0.293; 0.469) were found.

## 5. Discussion

The results of this Bayesian meta-analysis indicate that the consumption of hazelnuts has favorable effects on cholesterol serum levels. The diet intervention with hazelnut supplementation is consistently better than control diet at lowering serum LDL cholesterol concentrations and a trend toward the decrease of total cholesterol is found, while HDL cholesterol is not affected significantly.

The reason behind this positive effect on serum cholesterol levels could mainly be due to the lipid content of hazelnuts. The nutritional composition of hazelnuts shows a lipid pattern that differs compared to other types of nuts [37,38], though it most closely resembles that of almonds. Hazelnuts are the second richest source of monounsaturated fatty acids among nuts and are rich in vitamin E, phytosterols, l-arginine, polyphenols, folate and fiber. Hazelnuts fat composition contains 82%–83% MUFAs, mainly oleic acid (18:1), and 7.7%–8% of saturated fatty acids [11,12], with the highest unsaturated-saturated fatty acids ratio among nuts [37].

Similarly to what observed in olive oil, hazelnuts are very rich in monounsaturated fatty acids (82%–83% in hazelnuts; 79% in olive oil), with low content of omega-6 PUFA and of SFA [39]. This lipid pattern could explain most of the lowering effect of hazelnuts on LDL cholesterol as it happens with olive oil. As a matter of fact, the positive effect of olive oil on cholesterol serum levels has been clearly demonstrated in many studies and meta-analyses and represents one of the most efficacious components of the Mediterranean diet on decreasing LDL-C [40]. Anyhow, the obtained results suggest that additional components present in hazelnuts further reduce LDL-C concentrations beyond the effects theoretically predicted by equations based only on the fatty acid pattern [41,42].

The additional possible mechanisms that could be involved in the reduction of LDL cholesterol concentrations after hazelnut consumption may be due to various micronutrients and bioactive substances found in hazelnuts [11]. The first mechanism that could be involved is based on the antioxidant effect of hazelnuts because they are rich in vitamin E and tocopherols, phytosterols (mainly â-sitosterol) [10], magnesium, copper and selenium [12,13]. The antioxidant activity of hazelnuts may be due also to various phenolic compounds such as gallic acid, p-hydroxy benzoic acid, epicatechin, caffeic acid, sinapic acid, and quercetin [14]. Moreover, hazelnuts show a high content of l-arginine, which is a precursor of nitric oxide and could therefore contribute to the antiatherogenic effect of hazelnuts [35].

In addition, the high content of dietary fibers (8.1%) that could reduce the bioavailability of dietary cholesterol by means of a reduction of its intestinal absorption particularly on the basis of the soluble fiber component must be underlined [43]. Recently, it has been shown that the supplementation of hazelnut skin extract, which is rich in fiber and polyphenols, improves the plasma lipid profile in hamsters fed a high-fat diet [44].

The lowering effect of hazelnuts on LDL cholesterol serum concentration is similar to that found with a variety of nuts, particularly walnuts, almonds, peanuts and pecan nuts, and this underlines the need to support the consumption of mixed nuts to induce favorable effects on serum cholesterol and possibly prevent CVD [45,46]. Concerning the significant LDL cholesterol reduction, it has to be noted that the overall means of a daily dose of hazelnuts and duration of intervention among meta-analyzed studies are 38.7 g/day and 74.7 days, respectively. On the basis of this Bayesian meta-analysis, this represents the target hazelnut intervention in order to obtain an LDL cholesterol reduction. Obviously, this finding needs to be confirmed with additional studies, due to the small number of available trials useful for this meta-analysis and as a result of the larger daily amounts evidenced in most of the papers included in the systematic review (mean value: 45 g/day). As a matter of fact, major limitations of this meta-analysis are represented by the small number of available controlled studies and by the small number of subjects included in the analysis. Moreover, it is notable that two out of the three study groups included in the meta-analysis came from a single research group (Tey et al.) [7,8]. In order to clarify these aspects and to achieve additional information that could be of relevant clinical importance, such as data on inflammatory markers, additional studies with a large sample size are warranted.

Concerning the meta-analysis limitations, forest plots have not been represented because the studies did not provide confidence intervals and standard error of the MDΔs. Moreover, it has not been possible to perform a meta-regression or subgroup analysis (e.g., concerning a dosage indicator or follow-up period) because of the poor number of studies (*n* = 3). Accounting for this, we did not provide an index of the studies’ heterogeneity [47].

Even though the data regarding apolipoproteins could not be involved in the Bayesian meta-analysis due to the poor number and quality of the studies, the systematic review of the available values showed results which are in line with the cholesterol changes demonstrated by the meta-analytic evaluation. As a matter of fact, ApoB showed a significant decrease in three out of six studies and Apo A appeared unchanged in four studies and increased in two. These data as well as the results obtained with the systematic review regarding TC, TG and BMI could be influenced by the different baseline values and by the different dose of hazelnut supplementation.

Regarding the significant reduction of LDL cholesterol, the meta-analyzed studies showed a mean daily intake of 38.7 g of hazelnuts, i.e., 248 kcalories (1038 kJ), and a mean duration of intervention of 74.7 days. Even in presence of this high and prolonged caloric supplementation, the Bayesian meta-analysis of the available data showed an absence of body weight change (evaluated by means of BMI data) after hazelnut intervention. This result is very favorable, due to the negative effects of body weight gain on CVD, and reiterates what was observed after walnut supplementation [48].

## 6. Conclusions

In conclusion, a hazelnut-enriched diet decreases low-density lipoprotein cholesterol (LDL-C) in a significant way and shows a trend toward reduction of total cholesterol, without decreasing high-density lipoprotein cholesterol (HDL-C); while triglycerides and body mass index (BMI) remain substantially unchanged. These data show a potentially favorable effect on cardiovascular disease (CVD) prevention and suggest the need for further research with long-term intervention studies and large study groups in order to confirm and subsequently promote the consumption of hazelnuts to benefit the physiological cholesterol serum pattern.

## Figures and Tables

**Table 1:** Study characteristics.

First Author and Year (Reference)	Sample Size	Age (Years) (Mean ± SD)	BMI (kg/m^2^) or Weight (kg)	Sample Gender Distribution	Baseline Lipid Values * (Mean ± SD or 95%CI)	Study Design (Level of Evidence)	Trial Duration (Days)	Hazelnut Diet	Control Diet
Tey et al., 2015 [9]	39	21.4 ± 2.7	BMI: 25.00 ± 1.10 Weight: 73.9 ± 1.2	Maori: 20 males: 8 females: 12 European: 19 males: 5 females: 14	Maori group: TC: 4.14 (3.77–4.54) HDL-C: 1.16 (1.06–1.26) LDL-C: 2.46 (2.17–2.79) TG: 1.01 (0.84–1.22) Apo A: 1.51 (1.41–1.61) Apo B: 0.71 (0.63–0.80) European group: TC: 3.96 (3.63–4.31) HDL-C: 1.16 (1.01–1.32) LDL-C: 2.28 (2.01–2.59) TG: 0.96 (0.81–1.13) Apo A: 1.51 (1.39–1.66) Apo B: 0.65 (0.57–0.74)	Randomized controlled trials (level 2)	28	30 g/day	-
Tey et al., 2011 [6]	46	49.9 ± 9.4	BMI: 25.90 ± 3.50 Weight: 74.4 ± 13.1	males: 19 females: 27	TC: 5.88 ± 0.67 HDL-C: 1.21 ± 0.35 LDL-C: 4.01 ± 0.67 TG: 1.43 ± 0.63 Apo A: 1.78 ± 0.28 Apo B: 1.05 ± 0.21	Randomized cross-over (level 2)	84	30 g/day	-
Durak et al., 1999 [4]	30	18.5 ± NR	Weight: 68.7 ± 9.2	males: 18 females: 12	TC: 3.40 ± 0.51 HDL-C: 1.03 ± 0.26 LDL-C: 1.96 ± 0.51 TG: 0.87 ± 0.29	No randomized clinical trial (level 3)	30	69 g/day	-
Damavandi et al., 2013 [35]	48	55.68 ± 7.74	Hazelnut group BMI: 28.47 ± 3.57 Weight: 72.13 ± 10.27 Control group BMI: 28.18 ± 3.55 Weight: 71.98 ± 9.58	Hazelnut group: 23 males: 7 females: 16 Control group: 25 males: 8 females: 17	Hazelnut group: TC: 4.12 ± 0.95 HDL-C: 1.14 ± 0.21 LDL-C: 2.18 ± 0.75 TG: 1.75 ± 0.96 Control group: TC: 3.62 ± 0.76 HDL-C: 1.04 ± 0.20 LDL-C: 1.94 ± 0.72 TG: 1.41 ± 0.65	Randomized parallel study (level 2)	56	29 g/day	sel- selected diet
Orem et al., 2013 [36]	21	44.6 ± 10.4	BMI: 27.40 ± 3.11 Weight: 81.0 ± 14.1	males: 18 females: 3	TC: 5.77 ± 0.58 HDL-C: 1.12 ± 0.17 LDL-C: 4.00 ± 0.68 TG: 1.65 (1.12–2.18) Apo A: 1.31 ± 0.00 Apo B: 1.15 ± 0.00	Double control sandwich model (level 3)	56	49–86 g/day	-
Mercanligil et al., 2007 [5]	15	48 ± 8	BMI: 25.90 ± 1.7 Weight: 74.2 ± 5.1	males: 15 females: 0	TC: 5.86 ± 0.61 HDL-C: 1.13 ± 0.29 LDL-C: 3.80 ± 0.53 TG: 2.02 ± 1.51 Apo A: 1.32 ± 0.15 Apo B: 1.28 ± 0.22	Single group study (level 3)	56	40 g/day	-
Yücesan et al., 2010 [10]	21	28 ± 5	BMI: 23.89 ± 3.52 Weight: 64.52 ± 11.5	males: 8 females: 13	TC: 4.21 ± 0.59 HDL-C: 1.38 ± 0.32 LDL-C: 2.81 ± 0.66 TG: 1.01 ± 0.67 Apo A: 1.35 ± 0.18 Apo B: 0.78 ± 0.20	Single group study (level 3)	30	49–86 g/day	-
Tey et al., 2013 [8]	107	42.5 ± 12.4	Hazelnut group 30 g BMI: 30.70 ± 4.70 Weight: 86.2 ± 11.8 Hazelnut group 60 g: BMI: 30.90 ± 6.0 Weight: 92.0 ± 19.6 Control group: BMI: 30.40 ± 4.50 Weight: 88.7 ± 16.7	Hazelnut group 30 g: males: 13 females: 20 Hazelnut group 60 g: males: 17 females: 20 Control group: males: 16 females: 21	Hazelnut group 30 g: TC: 4.92 ± 1.00 HDL-C: 1.26 ± 0.25 LDL-C: 3.07 ± 0.86 TG: 1.29 ± 0.44 Apo A: 1.60 ± 0.03 Apo B: 0.88 ± 0.04 Hazelnut group 60 g: TC: 4.93 ± 1.01 HDL-C: 1.20 ± 0.32 LDL-C: 3.05 ± 0.96 TG: 1.49 ± 0.70 Apo A: 1.56 ± 0.05 Apo B: 0.89 ± 0.04 Control group: TC: 4.93 ± 1.00 HDL-C: 1.32 ± 0.33 LDL-C: 3.03 ± 0.86 TG: 1.27 ± 0.40 Apo A: 1.67 ± 0.04 Apo B: 0.87 ± 0.04	Randomized controlled parallel (level 2)	84	30 or 60 g/day	no nuts
Tey et al., 2011 [7]	61	37.5 ± 14	Hazelnut group: BMI: 24.60 ± 2.80 Weight: 72.0 ± 11.1 Control group: BMI: 22.90 ± 2.80 Weight: 67.3 ± 9.5	Hazelnut group: 32 males: 15 females: 17 Control group: 29 males: 12 females: 17	Hazelnut and control group: TC: 4.79 ± 0.95 HDL-C: 1.32 ± 1.30 LDL-C: 2.94 ± 0.84 TG: 0.98 ± 1.48	Randomized controlled parallel (level 2)	84	42 g/day	no additional food

BMI: Body Mass Index; TC: Total Cholesterol; HDL-C: High-Density Lipoprotein Cholesterol; LDL-C: Low-Density Lipoprotein Cholesterol; TG: Triglyceride; Apo A: Apolipoprotein A; Apo B: Apolipoprotein B. TC, HDL, LDL, TG are in mmol/L, ApoA, ApoB in g/L. sd: standard deviation; NR: not recorded.

**Table 2:** Inclusion and exclusion criteria of studies.

First Authors, Year (Reference)	Inclusion Criteria	Exclusion Criteria
Tey et al., 2015 [9]	Free-living Māori and European males and females, aged above 18 years living in Dunedin and surrounding areas.	Allergies or intolerances to nuts, medication known to affect blood lipid levels, familial or secondary hyperlipidemia, condition known to affect blood cholesterol levels, smoker, pregnant or lactating.
Tey et al., 2011 [6]	Healthy males or females aged between 18 and 65 years and: TC > 44.8 mmol/L and <8.0 mmol/L	Asthma, food allergies, familial hyperlipidemia, a chronic disease, or cholesterol-lowering medication or medication known to affect blood lipid concentrations.
Durak et al., 1999 [4]	healthy medical students aged 18 to 19 years	None
Damavandi et al., 2013 [36]	Previously diagnosed with type 2 diabetes based on FBS >126 mg/dL or 2-h blood sugar ≥200 mg/dL, serum TGs <400 mg/dL, body mass index (BMI) ≤35 kg/m^2^, Hemoglobin-A1C (HbA1C) <9%, serum LDL-C <200 mg/dL, and blood pressure ≤160/90 mmHg	Any known allergies to nuts, insulin therapy, cigarette smoking, history of stroke, heart disease or thyroid disorders, diabetic nephropathy or retinopathy, or following vegetarian or weight-loss diets up to 2 months before the study. Those patients who had consumed nuts more than 2 times/week and changed their medications (type or dosage) up to 2 months before the study were also excluded. All subjects were taking hypoglycemic agents.
Orem et al., 2013 [35]	Serum cholesterol level greater than 200 mg/dL with or without triacylglycerol greater than 150 mg/dL	On a medication or supplementation known to alter lipid metabolism
Mercanligil et al., 2007 [5]	Hypercholesterolemic (>200 mg/dL) adult males aged 33–59 years were recruited voluntarily from the staff of the Hacettepe University	Patients with TAG levels above 300 mg/dL were excluded from the study. All subjects were required not to be obese, be non-smokers and non-alcoholics, free of dietary restrictions/food allergies and not taking medications known to alter plasma lipids
Yücesan et al., 2010 [10]	Normolipidemic healthy subject among highly educated university staff members.	Systemic illness (diabetes mellitus, liver or kidney disease, or hypertension) or history of allergy to hazelnut. Also, individuals who consume alcohol or smoke were excluded. One of the exclusion criteria was the consumption of nuts, nut butters, or nuts oil more than once per week
Tey et al., 2013 [8]	Aged between 18 and 65 year inclusive, with a BMI 25 kg/m^2^	Asthma, allergies, or aversion to nuts; familial hyperlipidemia; major chronic disease; or inflammatory diseases such as Crohn’s or celiac disease. Current smokers, pregnant or breastfeeding women, and people who were participating in weight-loss programs or taking medications known to affect inflammatory markers were also excluded
Tey et al., 2011 [7]	Healthy males or females aged between 18 and 65 years	People with BMI ≥ 30 kg/m^2^, people who have asthma, women who are pregnant or breastfeeding, people with a chronic disease such as cancer, heart disease, or diabetes, and people with food allergies or food aversions

**Table 3:** Mean ∆-change* from baseline in hazelnut-enriched diet groups.

First Author, Year (Reference)	BMI (kg/m^2^) Mean Change (*p*-Value)	Body Weight (kg) Mean Change (*p*-Value)	TC (mmol/L) Mean Change (*p*-Value)	HDL-C (mmol/L) Mean Change (*p*-Value)	LDL-C (mmol/L) Mean Change (*p*-Value)	TG (mmol/L) Mean Change (*p*-Value)	APO-A (g/L) Mean Change (*p*-Value)	APO-B (g/L) Mean Change (*p*-Value)
Tey et al., 2015 [9]	0.00 (*p* = 0.938)	0.15 (*p* = 0.456)	0.00 (*p* = 0.983)	0.025 (*p* = 0.128)	−0.035 (*p* = 0.556)	0.005 (*p* = 0.834)	0.035 (*p* = 0.114)	−0.015 (*p* = 0.371)
Tey et al., 2011 [6]	0.01 (*p* = 0.822)	0.04 (*p* = 0.813)	−0.19 (*p* < 0.001)	0.03 (*p* = 0.023)	−0.22 (*p* < 0.001)	−0.01 (*p* = 0.725)	0.01 (*p* = 0.749)	−0.04 (*p* = 0.002)
Durak et al., 1999 [4]	NR	0.50 (*p* > 0.05)	−0.20 (*p* < 0.005)	0.09 (*p* < 0.05)	−0.36 (*p* < 0.0005)	0.21 (*p* < 0.001)	NR	NR
Damavandi et al., 2013 [36]	−0.55 (*p* > 0.05)	−0.66 (*p* > 0.05)	−0.12 (*p* > 0.05)	−0.06 (*p* > 0.05)	0.03 (*p* > 0.05)	−0.30 (*p* > 0.05)	NR	NR
Orem et al, 2013 [35]	−0.50 (*p* < 0.05)	−1.90 (*p* < 0.05)	−0.47 (*p* < 0.05)	0.07 (*p* < 0.05)	−0.25 (*p* < 0.05)	−0.27 (*p* < 0.05)	0.15 (*p* < 0.05)	−0.03 (*p* > 0.05)
Mercanligil et al., 2007 [5]	−0.10 (*p* > 0.05)	−0.20 (*p* > 0.05)	0.03 (*p* = 0.064)	0.15 (*p* = 0.001)	0.10 (*p* = 0.448)	−0.45 (*p* = 0.006)	0.04 (*p* = 0.557)	−0.07 (*p* = 0.01)
Yücesan et al., 2010 [10]	0.01 (*p* = 0.166)	0.21 (*p* = 0.166)	−0.36 (*p* < 0.001)	0.06 (*p* = 0.086)	−0.21 (*p* = 0.008)	−0.13 (*p* = 0.131)	0.06 (*p* = 0.005)	−0.07 (*p* = 0.005)
Tey et al., 2013 (30 g/day) ^ [8]	0.00 (*p* > 0.05)	0.00 (*p* > 0.05)	−0.14 (*p* > 0.05)	0.04 (*p* > 0.05)	−0.14 (*p* > 0.05)	−0.1 (*p* > 0.05)	0.03 (*p* > 0.05)	−0.03 (*p* > 0.05)
Tey et al., 2013 (60 g/day) ^ [8]	0.00 (*p* > 0.05)	0.2 (*p* > 0.05)	−0.13 (*p* > 0.05)	0.00 (*p* > 0.05)	−0.09 (*p* > 0.05)	−0.08 (*p* > 0.05)	0.01 (*p* > 0.05)	−0.02 (*p* > 0.05)
Tey et al., 2011 [7]	0.28 (*p* = 0.001)	0.83 (*p* = 0.001)	−0.06 (*p* = 0.398)	1.02 (*p* = 0.325)	−0.09 (*p* = 0.144)	0.99 (*p* = 0.349)	NR	NR

* The mean ∆-changes are post-pre. BMI: Body Mass Index; TC: Total Cholesterol; HDL-C: High-Density Lipoprotein Cholesterol; LDL-C: Low-Density Lipoprotein Cholesterol; TG: Triglyceride; Apo A: Apolipoprotein A; Apo B: Apolipoprotein B; NR: not recorded. ^ In Tey et al. (2013) [8] were administered two hazelnut dosages, 30 g/day and 60 g/day.

**Table 4:** Study quality and risk of bias assessment.

First Author, Year (Reference)	Sequence Generation	Allocation Concealment	Blinding	Blinding of Outcome Assessment	Incomplete Outcome Data	Selective Reporting	Overall Quality
Tey et al., 2015 [9]	+	+	-	-	+	+	Good
Tey et al., 2011 [6]	+	+	-	-	+	+	Good
Durak et al., 1999 [4]	-	-	-	-	+	+	Poor
Damavandi et al., 2013 [36]	-	+	-	-	+	+	Fair
Orem et al., 2013 [35]	-	+	-	-	+	+	Fair
Mercanligil et al., 2007 [5]	-	+	-	-	+	+	Fair
Yücesan et al., 2010 [10]	-	+	-	-	+	-	Fair
Tey et al., 2013 [8]	+	+	-	-	+	+	Good
Tey et al, 2011 [7]	+	+	-	-	+	+	Good

+: presence; -: absence.

**Table 5:** Meta-analysis data.

First Author, Year (Reference)	Total Cholesterol (mmol/L)			HDL-C (mmol/L)			LDL-C (mmol/L)			Triglycerides (mmol/L)			BMI (kg/m^2^)		
	Hazelnut Diet	Control Diet	MD Δ-changes (SD)	Hazelnut Diet	Control Diet	MD Δ-changes (SD)	Hazelnut Diet	Control Diet	MD Δ-changes (SD)	Hazelnut Diet	Control Diet	MD Δ-changes (SD)	Hazelnut Diet	Control Diet	MD Δ-changes (SD)
	t_0_ (SD)	t_0_ (SD)		t_0_ (SD)	t_0_ (SD)		t_0_ (SD)	t_0_ (SD)		t_0_ (SD)	t_0_ (SD)		t_0_ (SD)	t_0_ (SD)	
	t_1_ (SD)	t_1_ (SD)		t_1_ (SD)	t_1_ (SD)		t_1_ (SD)	t_1_ (SD)		t_1_ (SD)	t_1_ (SD)		t_1_ (SD)	t_1_ (SD)	
	Δ-changes (SD)	Δ-changes (SD)		Δ-changes (SD)	Δ-changes (SD)		Δ-changes (SD)	Δ-changes (SD)		Δ-changes (SD)	Δ-changes (SD)		Δ-changes (SD)	Δ-changes (SD)	
Damavandi et al., 2013 [36]	4.12 (0.95)	3.62 (0.76)	NR	1.14 (0.21)	1.04 (0.20)	NR	2.18 (0.75)	1.94 (0.72)	NR	1.75 (0.96)	1.41 (0.65)	NR	28.47 (3.57)	28.18 (3.55)	NR
	4.00 (0.95)	3.50 (0.81)		1.08 (0.17)	0.95 (0.19)		2.21 (0.72)	1.90 (0.75)		1.45 (0.80)	1.40 (0.95)		27.92 (3.57)	28.05 (3.64)	
	−0.12 (NR)	−0.12 (NR)		−0.06 (NR)	−0.09 (NR)		0.03 (NR)	−0.04 (NR)		−0.30 (NR)	−0.01 (NR)		−0.55 (NR)	−0.13 (NR)	
Tey et al., 2011 [7]	4.79 (0.95)	4.79 (0.95)	NR	1.32 (1.30)	1.32 (1.30)	NR	2.94 (0.84)	2.94 (0.84)	NR	0.98 (1.48)	0.98 (1.48)	NR	23.76 (2.99)	23.76 (2.99)	NR
	4.73 (NR)	4.89 (NR)		2.34 (NR)	2.32 (NR)		2.85 (NR)	3.03 (NR)		1.97 (NR)	2.01 (NR)		24.04 (NA)	23.90 (NA)	
	−0.06 (0.07)	0.10 (0.07)		1.02 (1.02)	1.00 (1.02)		−0.09 (0.06)	0.09 (0.07)		0.99 (1.04)	1.03 (1.05)		0.28 (0.08)	0.14 (0.18)	
Tey et al., 2013 (30 g/day) [8]	4.92 (0.17)	4.93 (0.17)	NR	1.26 (0.04)	1.32 (0.05)	NR	3.07 (0.15)	3.03 (0.14)	NR	1.29 (0.08)	1.27 (0.07)	NR	30.70 (0.81)	30.40 (0.74)	NR
	4.78 (0.16)	4.91 (0.16)		1.30 (0.04)	1.34 (0.06)		2.93 (0.15)	3.05 (0.15)		1.19 (0.07)	1.13 (0.05)		30.70 (0.81)	30.40 (0.78)	
	−0.14 (NR)	−0.02 (NR)		0.04 (NR)	0.02 (NR)		−0.14 (NR)	0.02 (NR)		−0.10 (NR)	−0.14 (NR)		0 (NA)	0 (NR)	
Tey et al., 2013 (60 g/day) [8]	4.93 (0.17)	4.93 (0.17)	NR	1.20 (0.05)	1.32 (0.05)	NR	3.05 (0.16)	3.03 (0.14)	NR	1.49 (0.12)	1.27 (0.07)	NR	30.90 (0.99)	30.40 (0.74)	NR
	4.80 (0.16)	4.91 (0.16)		1.20 (0.05)	1.34 (0.06)		2.96 (0.15)	3.05 (0.15)		1.41 (0.09)	1.13 (0.05)		30.90 (0.97)	30.40 (0.78)	
	−0.13 (NR)	−0.02 (NR)		0 (NR)	0.02 (NR)		−0.09 (NR)	0.02 (NR)		−0.08 (NR)	−0.14 (NR)		0 (NR)	0 (NR)	

HDL-C: High-Density Lipoprotein Cholesterol; LDL-C: Low-Density Lipoprotein Cholesterol; BMI: Body Mass Index; t_0_: pre value; t_1_: post value; MD: mean difference; Δ-change from baseline (post-pre); SD: standard deviation (or standard error); NR: not recorded.

**Table 6:** Results of the Bayesian meta-analysis.

Outcome	Parameters	Model 1 *				Model 2 **				Model 3 ***			
		Median	SD	95%HPD	MC Error	Median	SD	95%HPD	MC Error	Median	SD	95%HPD	MC Error
Total cholesterol	μ	−0.127	0.084	−0.284; 0.014	0.000	−0.127	0.173	−0.474; 0.215	0.001	−0.126	0.147	−0.418; 0.159	0.001
	τ^2^	0.001	0.270	0.000; 0.040	0.001	0.007	0.310	0.000; 0.447	0.004	0.006	0.204	0.000; 0.264	0.003
	ρ	0.863	0.452	−0.429; 0.999	0.003	0.756	0.522	−0.646; 0.999	0.004	0.778	0.512	−0.611; 0.999	0.005
	DIC	3.1				5.9				5.5			
HDL-C	μ	0.002	0.082	−0.140; 0.147	0.000	0.003	0.171	−0.341; 0.361	0.001	0.005	0.150	−0.297; 0.321	0.001
	τ ^2^	0.001	0.214	0.000; 0.044	0.001	0.007	0.303	0.000; 0.460	0.006	0.008	0.213	0.000; 0.309	0.006
	ρ	0.493	0.564	−0.776; 0.999	0.003	0.351	0.580	−0.831; 0.999	0.004	0.352	0.580	−0.829; 0.999	0.004
	DIC	0.1				1.4				1.2			
LDL-C	μ	−0.150	0.088	−0.308; −0.003	0.000	−0.149	0.179	−0.478; 0.210	0.001	−0.150	0.155	−0.437; 0.165	0.001
	τ^2^	0.001	0.347	0.000; 0.045	0.001	0.009	0.319	0.000; 0.442	0.004	0.008	0.228	0.000; 0.292	0.004
	ρ	0.747	0.490	−0.574; 0.999	0.003	0.646	0.537	−0.705; 0.999	0.004	0.648	0.531	−0.689 0.999;	0.004
	DIC	0.1				2.5				2.2			
Triglycerides	μ	0.045	0.141	−0.195; 0.269	0.001	0.040	0.293	−0.590; 0.627	0.001	0.041	0.243	−0.510; 0.495	0.001
	τ^2^	0.002	0.968	0.000; 0.118	0.004	0.029	0.537	0.000; 1.201	0.006	0.023	0.396	0.000; 0.708	0.003
	ρ	0.508	0.560	−0.767; 0.999	0.003	0.375	0.577	−0.821; 0.999	0.004	0.391	0.577	−0.816; 0.999	0.004
	DIC	3.2				4.7				4.5			
BMI	μ	0.062	0.245	−0.293; 0.469	0.001	0.062	0.419	−0.867; 0.979	0.002	0.062	0.347	−0.665; 0.803	0.002
	τ^2^	0.003	2.594	0.000; 0.286	0.007	0.078	0.716	0.000; 2.072	0.006	0.051	0.543	0.000; 1.165	0.006
	ρ	0.936	0.381	−0.156; 0.999	0.003	0.862	0.470	−0.471; 0.999	0.004	0.884	0.447	−0.405; 0.999	0.004
	DIC	12.1				15.7				14.8			

**HDL-C**: High-Density Lipoprotein Cholesterol: **LDL-C**: Low-Density Lipoprotein Cholesterol; **BMI**: Body Mass Index; μ: pooled (overall) mean difference—changes across studies; τ^2^: between-study variance; ρ: within-subject correlation between post and pre values; **DIC**: Deviance Information Criterion; **SD**: Standard Deviation; **95%HPD**: 95% Highest Posterior Density interval [2.5%; 97.5%]; **MC error**: Monte Carlo error gives an estimate of the Monte Carlo standard error of the mean; * **Model 1**: (τ^*2*^)^−1^ prior—Gamma(0.001, 0.001); ** **Model 2**: τ prior—Uniform(0, 100); *** **Model 3**: τ prior—Uniform(0, 2). In **bold** the significant results: MD∆ = 0 is not included in 95%HPD.

## Appendix. Jags Code of the Statistical Model for Bayesian Meta-Analysis

		**Model 1: (τ^2^)^−1^ prior—Gamma(0.001, 0.001)**
		model{
		## Likelihood
		for (i in 1:k) {
		# Variance of change in Treatment group (post-pre diff)
		vdT[i] <- vpostT[i] + vpreT[i] - (2*rho*sqrt(vpostT[i]*vpreT[i]))
		# Variance of change in Control group (post-pre diff)
		vdC[i] <- vpostC[i] + vpreC[i] - (2*rho*sqrt(vpostC[i]*vpreC[i]))
		# Variance of the difference between Treatment and Control (pooled variance)
		diff.var[i] <- ((nT[i]-1)*vdT[i] + (nC[i]-1)*vdC[i])/( nT[i]+nC[i]-2)
		w[i] <- 1/diff.var[i]
		diff[i] ~ dnorm(theta[i],w[i])
		theta[i] ~ dnorm(mu,prec)
		 }
		# 4th study that have Variance of the difference between Treatment and Control
		w4 <- 1/vd4
		diff4 ~ dnorm(theta4,w4)
		theta4 ~ dnorm(mu,prec)
		## Priors
		mu ~ dnorm(0.0,0.0001)
		prec ~ dgamma(0.0001,0.0001)
		tau2 <- 1/prec
		rho ~ dunif(-1,1)
		}
		**Model 2: τ prior ~ Uniform(0, 100)**
		model{
		## Likelihood
		for (i in 1:k) {
		# Variance of change in Treatment group (post – pre diff)
		vdT[i] <- vpostT[i] + vpreT[i] - (2*rho*sqrt(vpostT[i]*vpreT[i]))
		# Variance of change in Control group (post – pre diff)
		vdC[i] <- vpostC[i] + vpreC[i] - (2*rho*sqrt(vpostC[i]*vpreC[i]))
		# Variance of the difference between Treatment and Control (pooled variance)
		diff.var[i] <- ((nT[i]-1)*vdT[i] + (nC[i]-1)*vdC[i]) /(nT[i]+nC[i]-2)
		w[i] <- 1/diff.var[i]
		diff[i] ~ dnorm(theta[i],w[i])
		theta[i] ~ dnorm(mu,prec)
		}
		# 4th study that have Variance of the difference between Treatment and Control
		w4 <- 1/vd4
		diff4 ~ dnorm(theta4,w4)
		theta4 ~ dnorm(mu,prec)
		## Priors
		mu ~ dnorm(0.0,0.0001)
		prec <- 1/(tau * tau)
		rho ~ dunif(-1,1)
		tau ~ dunif(0,2)
		tau2 <- pow(tau,2)
		}
		**Model 3: τ prior ~ Uniform(0, 2)**
		model{
		## Likelihood
		for (i in 1:k) {
		# Variance of change in Treatment group (post-pre diff)
		vdT[i] <- vpostT[i] + vpreT[i] - (2*rho*sqrt(vpostT[i]*vpreT[i]))
		# Variance of change in Control group (post-pre diff)
		vdC[i] <- vpostC[i] + vpreC[i] - (2*rho*sqrt(vpostC[i]*vpreC[i]))
		# Variance of the difference between Treatment and Control (pooled variance)
		diff.var[i] <- ((nT[i]-1)*vdT[i] + (nC[i]-1)*vdC[i]) /(nT[i] + nC[i]-2)
		w[i] <- 1/diff.var[i]
		diff[i] ~ dnorm(theta[i],w[i])
		theta[i] ~ dnorm(mu,prec)
		}
		# 4th study that have Variance of the difference between Treatment and Control
		w4 <- 1/vd4
		diff4 ~ dnorm(theta4,w4)
		theta4 ~ dnorm(mu,prec)
		## Priors
		mu ~ dnorm(0.0,0.0001)
		prec <- 1/pow(tau,2)
		rho ~ dunif(-1,1)
		tau ~ dnorm(0,1)I(0,)
		tau2 <- pow(tau,2)
		
